# A Changing Paradigm: The Swift Expansion of Adult Living Donor Liver Transplantation for MASH Cirrhosis

**DOI:** 10.21203/rs.3.rs-10757904/v1

**Published:** 2026-08-28

**Authors:** Rei Matsumoto, Stephanie Silpe, Maria E. Cortes, Alexis Kim, Cheng Han Ng, Anh T. Bui, Garren Mongtomery, Brian J. Wentworth, Curt K. Argo, Nicolas M. Intagliata, Neeral L. Shah, Stephen H. Caldwell, Anya Mezina, Yazan Al-Adwan, Shawn Pelletier, Juan F. Guerra, Anita Sites, Mohammad S. Siddiqui, Zachary H. Henry

**Affiliations:** University of Virginia; University of Virginia; University of Virginia; Virginia Commonwealth University; National University, National University Hospital; Virginia Commonwealth University; University of Virginia; University of Virginia; University of Virginia; University of Virginia; University of Virginia; University of Virginia; University of Virginia; University of Virginia; University of Virginia; University of Virginia; University of Virginia; University of Virginia; University of Virginia

**Keywords:** living donor liver transplant, metabolic dysfunction associated steatohepatitis, alcohol associated liver disease, cirrhosis, liver transplant

## Abstract

Living donor liver transplantation (LDLT) provides a lifesaving option for patients with end-stage liver disease who may not be prioritized for deceased donor transplantation under the current model for end-stage liver disease (MELD)-based allocation system. Although prior studies have demonstrated a survival benefit for LDLT compared with remaining on the waitlist, limited data account for evolving liver disease epidemiology and donor characteristics in the United States.

**Methods:**

Using data from the Scientific Registry of Transplant Recipients/United Network of Organ Sharing (SRTR/UNOS), we conducted a retrospective cohort analysis of adult LDLT recipients and donors from 2000 to 2024. Trends were assessed using descriptive statistics and logistic regression.

**Results:**

A total of 7,686 LDLT recipients were identified. Indications included cholestatic liver disease (23%), hepatitis C virus (HCV) infection (16%), metabolic dysfunction–associated steatohepatitis (MASH) (15%), and alcohol-associated liver disease (ALD) (14%). MELD scores at LT varied by etiology, with higher scores in MASH and ALD. From 2000 to 2024, LDLT for MASH increased 5.4-fold (5.6% to 30.2%), while HCV declined sharply after 2014. LDLT for ALD remained stable despite increasing waitlist registrations. MASH recipients were more likely to receive grafts from younger related donors and less likely from older related donors or spouses compared with cholestatic liver disease.

**Conclusions:**

MASH is the fastest-growing indication for LDLT in the United States. These findings highlight limitations of MELD-based allocation and suggest a need to address donor eligibility barriers to improve equitable access to transplantation across liver disease etiologies.

## INTRODUCTION

End-stage liver disease (ESLD) due to decompensated cirrhosis is increasing in the United States, largely driven by metabolic dysfunction–associated steatohepatitis (MASH) and alcohol-related liver disease (ALD) ^1^ . Concurrently, indications for liver transplantation have expanded beyond traditional cirrhosis-related causes to include acute alcoholic hepatitis, intrahepatic cholangiocarcinoma, hepatocellular carcinoma (HCC) beyond Milan criteria, and select non-HCC malignancies such as metastatic colorectal cancer with isolated metastases to the liver and neuroendocrine tumors^2–4^. These expanded indications have reduced the availability of deceased donor organs for patients with decompensated cirrhosis, contributing to lower transplant rates and higher waitlist mortality^5^. Living donor liver transplantation (LDLT) has emerged as an important strategy to address patients in whom the mortality risk is not captured by the Model for End stage Liver Disease (MELD) score. LDLT provides a clear survival advantage over waiting for a deceased donor with recent registry data showing a survival benefit even among patients with lower MELD scores^6,7^.

Over the past two decades, the epidemiology of liver disease leading to transplantation in the United States has shifted markedly from hepatitis C virus (HCV) infection to MASH and ALD^1,8^. This transition reflects both the success of direct-acting antiviral therapy for HCV and the rising prevalence of metabolic syndrome, obesity, and hazardous alcohol use. Recent national registry data show that ALD is now the leading indication for liver transplantation in the U.S., followed closely by MASH, together accounting for more than 60% of new listings^1^.

Most existing evidence regarding living donor liver transplantation (LDLT) outcomes was generated in the pre–direct-acting antiviral era, when HCV was the dominant indication. As a result, limited contemporary data describe LDLT outcomes among patients with MASH and ALD. Understanding these evolving trends is critical as the burden of MASH and ALD continues to rise and increasingly defines the landscape of liver transplantation in the United States. To address these knowledge gaps, we utilized Scientific Registry of Transplant Recipients/United Network of Organ Sharing (SRTR/UNOS) data to evaluate changes in living donor liver transplantation in the United States over the past 25 years.

## METHODS

This retrospective analysis utilized data from the SRTR/UNOS to evaluate temporal trends and changing demographics of LDLT in the US. Briefly, the SRTR/UNOS database administers the Organ Procurement and Transplantation Network (OPTN) under contract with the U.S. Department of Health and Human Services. This deidentified, publicly available dataset includes information on all waitlist registrants and transplant recipients for solid organ transplantation in the United States since October 1987. The study was conducted in accordance with the Strengthening the Reporting of Observational Studies in Epidemiology (STROBE) guidelines for observational research^9^.

### Study Population

Using data from the SRTR/UNOS, this study conducted a retrospective analysis of all living donor and recipient pairs undergoing LDLT from January 1, 2000, to December 2, 2024. The analysis was restricted to adult recipients (age ≥ 18 years at the time of surgery) to focus on trends in the adult population. No additional exclusion criteria were applied beyond the age cut-off. For liver disease categorization, SRTR/UNOS registry codes were used for specific etiologies like HCV, primary biliary cirrhosis (PBC), primary sclerosing cholangitis (PSC), autoimmune hepatitis (AIH), MASH, etc. To more accurately capture cases of MASH, patients with a cryptogenic cirrhosis diagnosis who also had a BMI ≥ 30kg/m^2^ or diabetes were reclassified as having MASH cirrhosis as previously reported ^5^. Due to the relatively lower number of patients with PBC or PSC, they were combined together as cholestatic liver disease. For patients with HCC, the primary etiology of their liver disease was prioritized as the main diagnosis.

### Statistical Analysis

All collected clinical and demographic data of donors and recipients are summarized as median with interquartile range (IQR) or N (%). Trends over time were evaluated using univariate logistic regression (for binary outcome, such as percentage) or linear regression (for number of casses) with the calendar year being an independent variable. The temporal trends in LDLT were analyzed initially as the contribution of each liver disease to the total LDLT performed during that calendar year. During the study period, there have been substantial changes in the composition of waitlist registrants (decreasing viral hepatitis and increasing MASH and ALD). Therefore, to account for these spatial trends we analyzed the proportions of LDLT compared to total LT (LDLT +Deceased donor LT) for each etiology of cirrhosis. To better assess how changes in liver allocation policy influenced the utilization of living donor liver transplantation (LDLT), we evaluated era effects by segmenting the period from 2002 to 2024 according to the predominant allocation system in use: MELD, MELD-Na, and MELD 3.0. Because metabolic dysfunction–associated steatohepatitis (MASH) and alcohol-associated liver disease (ALD) collectively represent the majority of liver transplants performed in the United States, the analysis was restricted to these two indications to more clearly delineate how policy-era transitions shaped national LDLT patterns. Across group differences were compared using analysis of variance (ANOVA) for continuous variables and Chi-square test for proportions. For this analysis, we focused on MASH, ALD, HCV, cholestatic liver disease, and autoimmune hepatitis (AIH) as these are the most common indications for LDLT. A nominal p-value of 0.05 was considered statistically significant. All analyses were run in STATA 16.1 (StataCorp LLC, College Station, TX).

## RESULTS

The study cohort comprised 7,686 partial liver donors who underwent donation between 2000 and 2024 across participating U.S. centers. Baseline characteristics of the donor cohort are summarized in Table 1. Overall, donors were more likely to be female (52%) and non-Hispanic White (80.5%). The median donor age at the time of donation was 37 (IQR 29, 46) years, and the median body mass index (BMI) was 25.8 (23.1–28.7) kg/m^2^, with 15.4% meeting criteria for obesity. Donor comorbidity burden was low, as expected for a carefully selected living donor population. Baseline characteristics of LDLT recipients are also presented in Table 1. As an aggregate, the most common underlying etiologies of cirrhosis leading to LDLT were cholestatic liver diseases (PSC and PBC; 23%), hepatitis C virus (HCV) infection (16%), MASH (15%), and ALD (14%).

### Temporal Trends in Etiologies of Cirrhosis Requiring Living Donation

**Figure 1:**
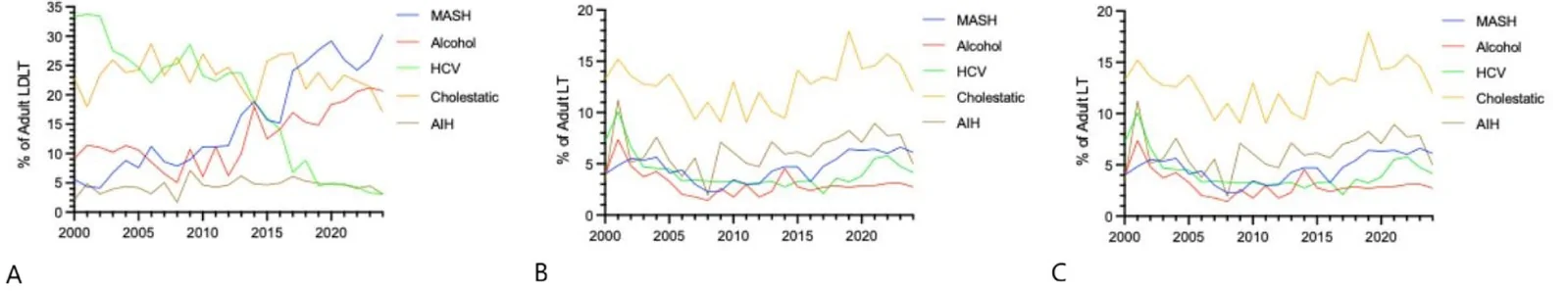
**A**: Temporal trend in adult living donor liver transplant (LDLT) demonstrates metabolic dysfunction associated steatohepatitis (MASH) as the most rapidly grown indication requiring liver transplant (LT) followed by alcohol associated liver disease (ALD). **B:** Adult LDLT as proportion of total adult LT demonstrates a similar trend with most rapid increase observed in patients with MASH cirrhosis. **C:**Contribution of hepatocellular carcinoma (HCC) as a co-diagnosis in patients receiving living donor liver transplant (LDLT). HCC is further categorized in those who received model of end stage liver disease (MELD) score exception compared to those who did not receive MELD exception.

The volume of LDLT declined between 2000 and 2010. However, since 2010, there has been a steady and sustained increase in the number of adult LDLT procedures performed in the United States; when evaluating the entire 25-year period, there was a significant upward trend overall (Supplemental Fig. 1) (β = 9.8, 95%CI: 4.3–15.2, p = 0.001). In the early 2000s, the predominant indications for LDLT were hepatitis C virus (HCV)–related cirrhosis, followed by cholestatic liver diseases, ALD and MASH (Fig. 1A). Over time, the etiologic landscape of liver disease requiring LDLT has shifted substantially. The most notable increase was observed among MASH recipients, whose representation in LDLT rose from 5.6% in 2000 to 30.2% in 2024, representing a 5.4-fold increase (OR 1.09, 95% CI: 1.08–1.1, p < 0.0001). In contrast, HCV-related cirrhosis as an indication for LDLT decline significantly over the past 25 years and accounted for only 3.1% of LDLT cases in 2024 (OR 0.89, 95%CI: 0.88–0.89, p < 0.0001). A modest but consistent rise in ALD cirrhosis was also observed, with rates increasing from 9.1% in 2000 to 20.6% in 2024, reflecting 2.3-fold increase (OR 1.05, 95%CI: 1.04–1.06, p < 0.0001).

To better contextualize these temporal trends, the distribution of LDLT compared to all LT for each etiology over the study duration was evaluated. Cholestatic liver disease, as an etiology of cirrhosis, was the indication with the highest percentage of patients receiving LDLT, followed by MASH and AIH (Fig. 1B). Percentage of LDLT trended down among all of the etiologies between 2000 to 2010 due to the decrease in overall LDLT case number. However, since 2010, percentage of LDLT increased over time significantly in MASH and cholestatic liver disease patients; there was a significant upward trend over the 25-year period (OR 1.05, 95%CI: 1.03–1.06, p < 0.0001, OR 1.02, 95%CI: 1.01–1.02, p < 0.001, respectively). The proportions of LDLT in ALD and HCV patients remained low after 2010; thus, from 2000 to 2024 overall, these etiologies showed either decreasing trend or no significant change (ALD: OR 0.99, 95%CI: 0.98–1.00, p = 0.051, HCV: OR 0.96, 95%CI: 0.95–0.97, p < 0.001). Percentage of LDLT among AIH patients also did not demonstrate a significant upward or downward trend over the 25-year period (OR 1.01, 95%CI: 0.99–1.03, p = 0.08). When patients were stratified by MELD era (MELD:2002–2015, MELD-Na:2016–2022, MELD 3.0: 2023–2024), a similar increase in the proportion of LDLT among NASH patients was observed in the MELD-Na era (OR 1.06, 95% CI: 1.02–1.09, p = 0.001), whereas no significant change was observed among ALD patients in any MELD era (MELD: p = 0.48, MELD-Na: p = 0.2, MELD 3.0: p = 0.2) (Supplemental Fig. 2).

To understand the interaction between HCC and LDLT, the number of HCC transplants performed both for MELD exception and non-MELD exception were quantified. Only 2.9% of patients who had HCC (MELD exception and non-MELD exception) received a LDLT (the rest underwent DDLT). Among all etiologies, patients with HCV had the highest proportion of HCC patients who qualified for MELD exception and subsequently received an LDLT (11.4%) (Fig. 1C).

### Changing in Demographics of Living Donors

**Figure 2:**
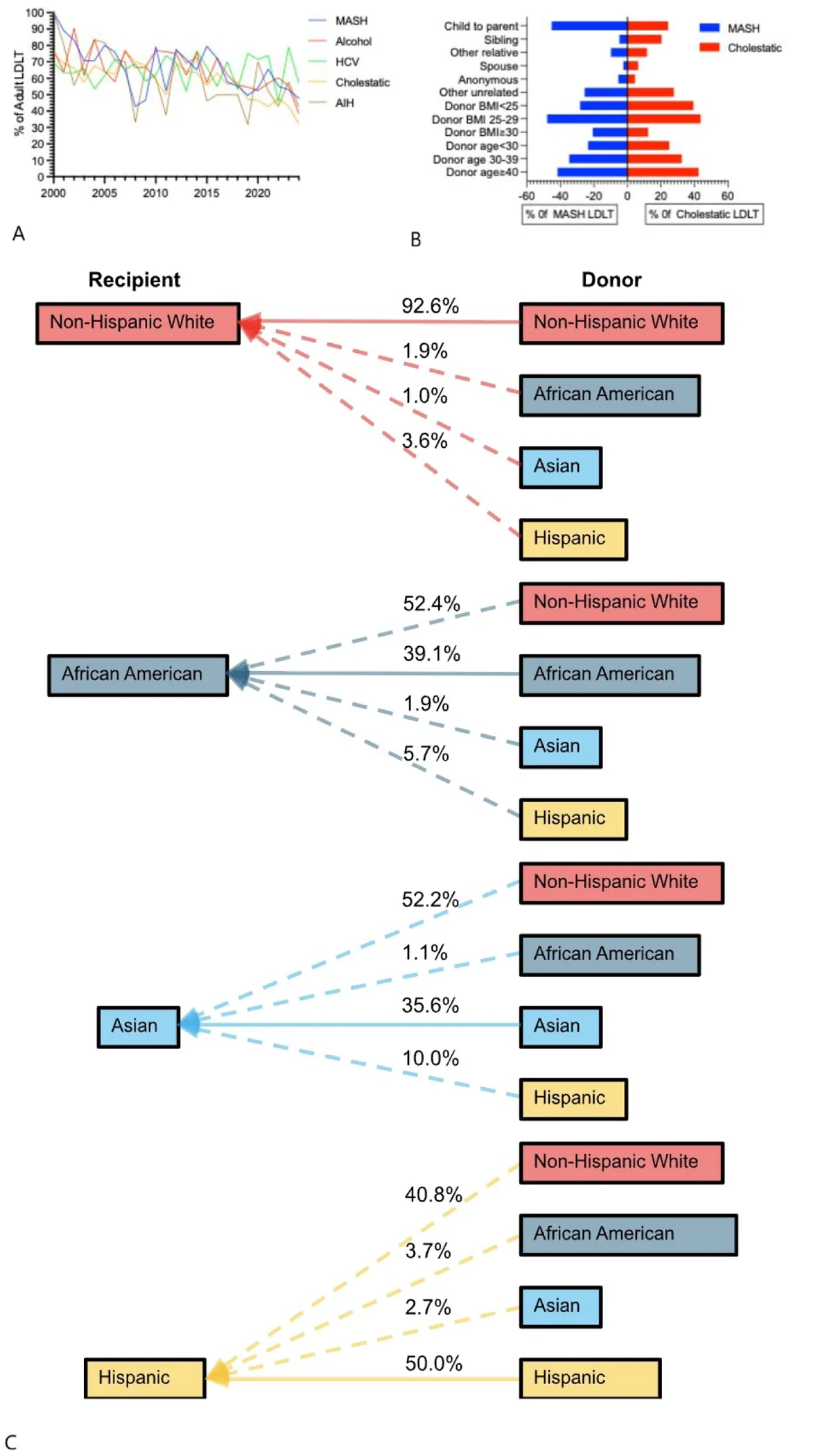
**A**: Proportions of related living donations in the US are decreasing. **B**: Comparison of living donor characteristics between recipients with metabolic dysfunction–associated steatohepatitis (MASH) and those with cholestatic liver disease. **C**: The figure depicts unrelated living donor liver transplants (LDLTs). Only 9.3% of all LDLTs are cross-racial donations, with non-Hispanic White recipients benefiting the most and Asian recipients the least. Percentages are calculated using the total number of unrelated LDLTs performed within each recipient ethnicity as the denominator and the number of donors of each ethnicity as the numerator.

Several demographic trends in living donations were observed over the study period. Overall, the proportion of living related donor transplants decreased over time (Fig. 2A). The most pronounced decline was noted in patients with MASH, in whom the proportion of living related donors [vs. living unrelated donors] decreased from 100% in 2000 to 47.8% in 2024 (OR 0.94, 95%CI: 0.92–0.96, p < 0.0001), accompanied by a corresponding rise in unrelated donations.

A more detailed comparison of related and unrelated donors among the two most common recipient etiologies of cirrhosis - cholestatic and MASH - demonstrated distinct differences in the composition of the donor pool. Recipients with cholestatic liver disease were more likely to receive living donations from siblings and spouses (20.4% vs 4.8%, p < 0.0001, 6.5% vs 2.5%, p < 0.0001, respectively), whereas recipients with MASH were more likely to receive living donations from their adult children (45.2% vs 24.4%, p < 0.0001) (Fig. 2B). Moreover, patients with MASH were more likely to receive donations from donors who had higher BMI when compared to donors for cholestatic liver disease recipients (BMI ≧ 30: 20.7% vs 12.3%, p < 0.0001, BMI 25–29: 47.9% vs 43.7%, p = 0.02) (Fig. 2B). The median BMI for related donors for MASH recipients was 27.1 (IQR 24.4, 29.5), whereas the median BMI for unrelated donors for MASH was 26.6 (IQR 23.6, 29.2).

Expectedly, living related donors tended to come from the same racial and ethnic background as their recipients, and the proportion of related living donation was consistent across racial groups (approximately 60–70%). However, notable patterns emerged when examining living unrelated donation (Fig. 2C). Cross-racial donations accounted for only 9.3% of all LDLT, underscoring how uncommon unrelated donation across racial groups is. Among unrelated donor–recipient pairs, Non-Hispanic White recipients received same-race donations in 92% of cases. In contrast, same-race unrelated donation among recipients from other racial and ethnic minority groups was substantially lower, ranging from approximately 35% to 50%. Given the overall small number of non-White LDLT recipients in the United States, these findings highlight the predominance of White-to-White living donor liver transplantation and the limited availability of racially concordant unrelated donors for minority patients.

### Comparison of Recipient Characteristics

**Figure 3:**
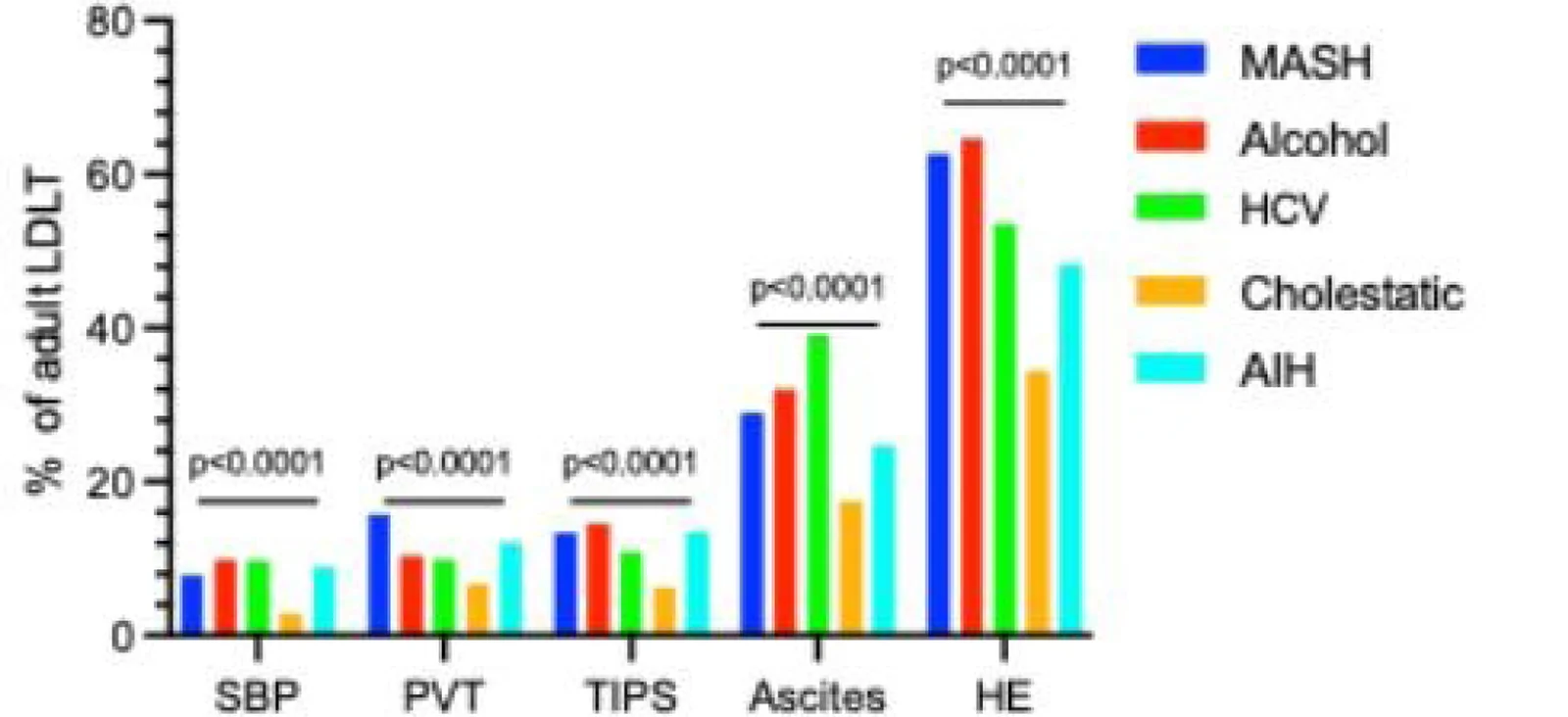
Complications of portal hypertension including spontaneous bacterial peritonitis (SBP), portal vein thrombosis (PVT), transjugular intrahepatic shunt (TIPS), ascites and hepatic encephalopathy among recipients of adult LDLT across the more common etiologies of recipient’s chronic liver disease. (MASH, metabolic dysfunction associated steatohepatitis; HCV, hepatitis C virus; AIH, autoimmune hepatitis)

The median MELD-Na score at the time of LT was higher among recipients with MASH (median MELD 16.5, IQR 12.7, 20.8) and ALD (median MELD 16.7, IQR 12.9, 21.5) compared to other etiologies (AIH: median MELD 15.7, IQR 12.2, 19.5; Cholestatic: median MELD 15.0, IQR 11.2, 19.7, and HCV: median MELD 15.4, IQR 11.9, 19.0). Complications of portal hypertension—including hepatic encephalopathy, ascites, portal vein thrombosis (PVT), and the need for transjugular intrahepatic portosystemic shunt (TIPS)—were less common in recipients with cholestatic liver disease compared to other etiologies (MASH, ALD, and HCV) (Fig. 3).

## DISCUSSION

Living donor liver transplantation (LDLT) represents an excellent alternative for patients with decompensated cirrhosis who may not have timely access to deceased donor transplantation due to lower MELD scores. Recent studies have demonstrated a significant survival benefit associated with LDLT compared to remaining on the waitlist. The present study expands upon prior work by examining temporal trends in living donation and characterizing the evolving demographics of living donors.

A key finding of this study is the dramatic increase in the number of LDLT performed for MASH cirrhosis. Between 2000 and 2024, the proportion of LDLT for MASH rose from 5% to nearly one in every six to seven LDLT performed in the United States ^1^ . While this trend may, in part, reflect the growing number of patients with MASH cirrhosis being listed for liver transplantation, published data and additional analyses from the present study suggest that other factors also contribute. To account for the proportional rise in MASH-related waitlist registrations, we examined the number of LDLT relative to the total number of liver transplants performed for each etiology. A major increase in the proportion of LDLT was observed for MASH over the study duration, underscoring that the rise in LDLT extends beyond changes in waitlist composition alone. This likely reflects the unique clinical trajectory of MASH cirrhosis, as these patients are often underserved by the MELD-based allocation system, leading to higher waitlist mortality and lower likelihood of receiving deceased donor offers^5^. Consequently, LDLT serves as an increasingly important strategy to improve access and outcomes among patients with MASH cirrhosis. While the proportion of living donor liver transplants (LDLT) relative to total liver transplants for cholestatic liver disease remained high, these rates did not significantly increase over time. This stability suggests that the evolving landscape of chronic liver disease—marked by rising rates of ALD and MASH among waitlist registrants—had minimal impact on LDLT trends for cholestatic etiologies.

Alcohol-associated liver disease represents the other major indication for liver transplantation in the United States and, together with MASH, accounts for the vast majority of procedures performed. In contrast to MASH, however, LDLT for ALD has remained relatively stable since 2000 as a proportion of total LT, even showing decreasing trend. This stability persists despite rising waitlist registrations for ALD and, more recently, an increase in transplants for acute alcohol-associated hepatitis ^10^ . In the context of prior literature, these divergent trajectories likely reflect the differing natural history of ALD, where patients—particularly those with alcoholic hepatitis—are more likely to receive deceased donor offers due to their high MELD scores, thereby reducing the need for LDLT. Furthermore, persistent societal stigma surrounding liver transplantation for ALD may contribute to the lower frequency of LDLT in this population, reflecting potential implicit biases that span both providers and potential living donors^11^.

In addition to recipient factors, several donor-related findings highlight the divergent nature of living donation in MASH. Prior studies have demonstrated a significantly higher prevalence of steatotic liver disease and advanced fibrosis among cohabitants of patients with MASH cirrhosis ^12^ . This likely contributes to the lower availability of spousal donors observed in the MASH cohort compared with cholestatic liver disease. Furthermore, the increased risk of metabolic dysfunction–associated steatotic liver disease (MASLD) and advanced fibrosis among first-degree relatives of patients with MASH cirrhosis may further constrain the pool of eligible sibling donors^13^. Consequently, a substantially high proportion of unrelated or non-direct donors was observed among recipients with MASH cirrhosis. These donors, who are less likely to share the same genetic or environmental risk factors, may be less frequently excluded due to elevated body weight or underlying hepatic steatosis, factors that commonly preclude donation among related or cohabitant individuals.

The present study provides novel data on temporal trends in LDLT in the US, highlighting the substantial impact of MASH. However, due to registry-based nature of the data, it is difficult to ascertain how institutional, provider, and patient related factors may impact these trends. The data reported to UNOS/SRTR represents donor-recipient pairs that have been approved for listing, thus, introducing a reporting bias where donors who are rejected from donation consideration are not registered in the database. Similarly, registry style data is not able to define why LDLT for certain disease types (i.e. ALD) or ethnicity (i.e. African Americans) are lower. Better understanding of provider, patient, and donor related factors for donors who are approved or rejected is essential to better understanding major limitations to living donations in the US.

In summary, this study demonstrates that MASH is the most rapidly growing indication for LDLT in the US. The rise in LDLT for MASH extends beyond waitlist composition, reflecting the limitations of MELD-based allocation and the increasing reliance on LDLT to improve access for these patients. In contrast, LDLT rates for alcohol-associated liver disease (ALD) and cholestatic disorders have showed a more gradual decrease or remained relatively stable. Donor-related factors, including higher rates of metabolic dysfunction among relatives and cohabitants, further constrain living donation for MASH. These findings underscore the need to identify and address barriers to donor eligibility and ensure equitable access to LDLT across liver disease etiologies.

## Supplementary Material

Supplementary Files

This is a list of supplementary files associated with this preprint. Click to download.

• LDLTvisual.docx

• Supplementalfig1.jpg

• Supplementalfig2.jpg

## Figures and Tables

**Table 1:** Baseline demographics of living donors and living donor liver transplant recipients

DEMOGRAPHICS	Living Donors (N = 7686)	Recipients (N = 7686)
	
		

Age (years)	37 (29, 46)	54 (45, 62)

Gender (%)		

Males	3,667 (48%)	4,147 (54%)

Females	4,019 (52%)	3,539 (46%)

Ethnicity (%)		

Non Hispanic Whites	6,185 (80.5%)	6,152 (80.0%)

Blacks	283 (3.7%)	277 (3.6%)

Hispanics	943 (12.3%)	961 (12.5%)

Asians	196 (2.6%)	225 (2.9%)

**LIVER DISEASE (%)**		

Alcohol	-	1,093 (14%)

MASH	-	1,141 (15%)

Cholestatic liver disease	-	1,783 (23%)

Hepatitis C virus	-	1,216 (16%)

**COMORBIDITES**		

Body mass index (kg/m2)	25.8 (23.1, 28.7)	26.4 (23.3, 30.1)

Obesity (%)	1,187 (15.4%)	1,966 (25.6%)

Diabetes (%)	-	1,659 (21.6%)

Hypertension (%)	-	567 (7.4%)

**LABORATORY PARAMETERS**		

Alanine aminotransferase (IU/L)	20 (15, 28)	52 (32, 88)

Bilirubin (mg/dL)	0.5 (0.4, 0.7)	2.4 (1.4, 4.5)

Albumin (mg/dL)	4.4 (4.2, 4.7)	2.9 (2.5, 3.4)

Sodium (mg/dL)	-	137 (135, 140)

Creatinine (mg/dL)	0.85 (0.7, 1.0)	0.86 (0.7, 1.1)

Model for End Stage Liver Disease Score	-	15.4 (11.4, 19.9)

MASH: metabolic dysfunction associated steatotic liver disease
